# Dual Function of the pUL7-pUL51 Tegument Protein Complex in Herpes Simplex Virus 1 Infection

**DOI:** 10.1128/JVI.02196-16

**Published:** 2017-01-03

**Authors:** Anna Albecka, Danielle J. Owen, Lyudmila Ivanova, Juliane Brun, Rukayya Liman, Laura Davies, M. Firoz Ahmed, Susanna Colaco, Michael Hollinshead, Stephen C. Graham, Colin M. Crump

**Affiliations:** Division of Virology, Department of Pathology, University of Cambridge, Cambridge, United Kingdom; University of California, Irvine

**Keywords:** UL51, UL7, focal adhesion, herpes simplex virus, human herpesviruses, tegument

## Abstract

The tegument of herpesviruses is a highly complex structural layer between the nucleocapsid and the envelope of virions. Tegument proteins play both structural and regulatory functions during replication and spread, but the interactions and functions of many of these proteins are poorly understood. Here we focus on two tegument proteins from herpes simplex virus 1 (HSV-1), pUL7 and pUL51, which have homologues in all other herpesviruses. We have now identified that HSV-1 pUL7 and pUL51 form a stable and direct protein-protein interaction, their expression levels rely on the presence of each other, and they function as a complex in infected cells. We demonstrate that expression of the pUL7-pUL51 complex is important for efficient HSV-1 assembly and plaque formation. Furthermore, we also discovered that the pUL7-pUL51 complex localizes to focal adhesions at the plasma membrane in both infected cells and in the absence of other viral proteins. The expression of pUL7-pUL51 is important to stabilize focal adhesions and maintain cell morphology in infected cells and cells infected with viruses lacking pUL7 and/or pUL51 round up more rapidly than cells infected with wild-type HSV-1. Our data suggest that, in addition to the previously reported functions in virus assembly and spread for pUL51, the pUL7-pUL51 complex is important for maintaining the attachment of infected cells to their surroundings through modulating the activity of focal adhesion complexes.

**IMPORTANCE** Herpesviridae is a large family of highly successful human and animal pathogens. Virions of these viruses are composed of many different proteins, most of which are contained within the tegument, a complex structural layer between the nucleocapsid and the envelope within virus particles. Tegument proteins have important roles in assembling virus particles as well as modifying host cells to promote virus replication and spread. However, little is known about the function of many tegument proteins during virus replication. Our study focuses on two tegument proteins from herpes simplex virus 1 that are conserved in all herpesviruses: pUL7 and pUL51. We demonstrate that these proteins directly interact and form a functional complex that is important for both virus assembly and modulation of host cell morphology. Further, we identify for the first time that these conserved herpesvirus tegument proteins localize to focal adhesions in addition to cytoplasmic juxtanuclear membranes within infected cells.

## INTRODUCTION

Herpesviridae comprises a family of evolutionarily old DNA viruses that are widely spread among vertebrates. Herpes simplex virus 1 (HSV-1) belongs to the Alphaherpesvirinae subfamily, which also includes the human pathogens HSV-2 and varicella-zoster virus (VZV). Infections with HSV-1 are commonly asymptomatic or cause relatively mild symptoms (e.g., cold sores). However, in immunocompromised individuals HSV-1 can lead to serious complications, such as herpes simplex encephalitis and keratitis, if infection spreads to the central nervous system or eye, respectively (1, 2). After primary infection of epithelial cells, HSV-1 spreads to sensory ganglia, where it establishes a lifelong latent infection followed by sporadic virus reactivation throughout the lifetime of the host (3).

Herpesvirus morphology has the characteristic presence of a complex protein layer between the viral capsid and the outer envelope. This layer, termed the tegument, contains many proteins (over 20 different viral proteins in HSV-1) harboring both structural and regulatory functions. Tegument proteins facilitate virus replication by regulating gene transcription, shutting off cellular protein synthesis, interacting with cellular transport machinery, and undermining innate immune responses (reviewed in reference 4). They also provide a scaffold for viral particle assembly, creating a network of interactions connecting the capsid with the viral envelope proteins (5, 6). Tegument proteins are often classified as “inner” or “outer” tegument proteins based on how tightly they are associated with the capsid after the envelope is removed. Little is known about the spatial organization of proteins within the tegument layer, and such a classification regarding inner versus outer tegument may not always reflect the actual protein location in the virion. However, recent advances in fluorescence microscopy imaging are starting to unravel the details of tegument organization (7, 8).

Here, we focus on the interaction and function of the HSV-1 tegument proteins pUL7 and pUL51. pUL7 is a 33-kDa protein that is expressed late during infection and conserved in all herpesviruses (9). Deletion of pUL7 from HSV-1 leads to a 10- to 100-fold decrease in production of infectious particles and a small-plaque phenotype (10). Interestingly, pUL7 was found to bind the adenine nucleotide translocator 2 protein that resides in mitochondria (10), but the precise role of this interaction in HSV-1 infection is not known. Decreased viral titer and small plaque size were also observed when the UL7 gene was deleted from pseudorabies virus (PRV), another member of the Alphaherpesvirinae subfamily (11). In this study, the authors observed a defect in secondary envelopment of nucleocapsids and less efficient secretion of assembled particles. In addition, the PRV UL7 deletion virus was moderately attenuated in mouse infection models and demonstrated a delay in neuroinvasion, highlighting a role of pUL7 in both *in vitro* and *in vivo* infections (11). pUL51 is a phosphoprotein that is also expressed during late stages of infection. The predicted molecular mass of pUL51 is 25.5 kDa, but slower-migrating bands of 27, 29, and 30 kDa are observed on reducing polyacrylamide gels (12). This can be explained by posttranslational modifications of pUL51, including palmitoylation of cysteine 9, which provides a lipid anchor, leading to protein association with cellular membranes (13). pUL51 is conserved among herpesviruses, and a role in secondary envelopment and maturation has been shown in HSV-1, PRV, and human cytomegalovirus (HCMV) (14–18). pUL51 deletion viruses replicate to lower titers, demonstrate decreased plaque size, and show increased intracellular accumulation of naked or partially assembled capsids, suggesting that pUL51 is involved in virus assembly (14, 16). More recently, during the time we were conducting our work on pUL7 and pUL51, another group reported that these two tegument proteins interact and pUL7 interaction with pUL51 is necessary for pUL7 recruitment to cellular membranes and into assembling virions (19).

In this report we show that pUL7 and pUL51 interact in cells in the presence or absence of other viral proteins and, furthermore, that these proteins directly interact using purified bacterially expressed pUL7 and pUL51. Deletion viruses lacking functional pUL7, pUL51, or both pUL7 and pUL51 have highly similar defects in replication and plaque size, suggesting that these proteins function as a complex during HSV-1 replication. Furthermore, we demonstrate that the pUL7-pUL51 complex, but not pUL7 or pUL51 alone, localizes to focal adhesion structures and that deletion of the pUL7-pUL51 complex results in a dramatic change in infected cell morphology.

## RESULTS

### pUL7 and pUL51 interact in transfected and infected cells.

**FIG 1:**
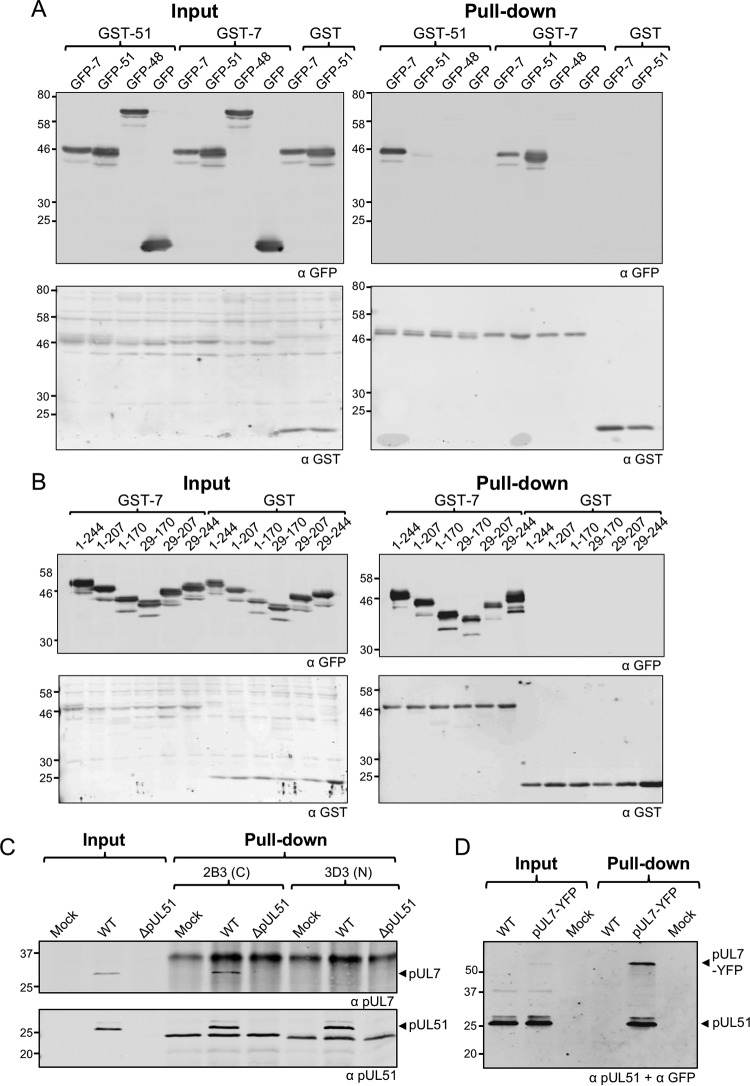
Interaction between pUL7 and pUL51 in transfected and infected cells. (A and B) 293T cells were cotransfected with plasmids carrying genes for GST-pUL51, GST-pUL7, or GST alone and GFP-pUL7, GFP-pUL51, GFP-pUL48, or GFP alone (A) or cotransfected with plasmids carrying genes for GST-pUL7 or GST alone, together with plasmids carrying genes for GFP-tagged full-length or truncated pUL51 (B). After 48 h, cells were lysed, and protein complexes were pulled down with glutathione-Sepharose beads. Proteins were separated by SDS-PAGE and immunoblotted, probing for GFP (top) and GST (bottom). (C) HaCaT cells were infected with WT or strain ΔpUL51 viruses and incubated for 16 h. Cell lysates were incubated with antibodies against pUL51, and the protein complexes were captured using protein A/G beads. The 3D3 antibody recognizes an epitope within the first 170 residues of pUL51, whereas 2B3 recognizes an epitope within residues 171 to 220 of pUL51. Samples were separated by SDS-PAGE and immunoblotted, probing for pUL7 (top) and pUL51 (bottom). (D) HaCaT cells were infected with WT or YFP-tagged pUL7 viruses and incubated for 16 h. Cell lysates were incubated with GFP-Trap–agarose beads to capture YFP-pUL7. Samples were separated by SDS-PAGE and immunoblotted, probing for pUL51 and YFP.

To investigate the interaction between pUL7 and pUL51 in the absence of other viral proteins, we first performed a range of glutathione *S*-transferase (GST) pulldown experiments with transfected 293T cells. Under these conditions, we were able to demonstrate interactions between pUL7 and pUL51 in both directions; GST-pUL51 pulled down coexpressed GFP-pUL7, and GST-pUL7 pulled down coexpressed GFP-pUL51 (Fig. 1A). Our results also suggest that these proteins may form higher-molecular-weight complexes, because GST-pUL51 pulled down GFP-pUL51 and GST-pUL7 pulled down GFP-pUL7, albeit with weaker signals than detected for the pUL7-pUL51 interactions. No interaction between pUL7 or pUL51 with the control viral tegument protein pUL48 (VP16) or free green fluorescent protein (GFP) was observed. Previous studies (13) and our own bioinformatics analysis (data not shown) suggested that pUL51 comprises a well-conserved N-terminal domain plus a less-conserved proline-rich C-terminal domain, the latter of which is predicted to be intrinsically unstructured. To investigate the pUL7-pUL51 interaction in more detail, we constructed a panel of pUL51 truncations that could be expressed as GFP fusion proteins. All the GFP-pUL51 constructs we tested retained pUL7 binding (Fig. 1B). These results suggest that a core domain of pUL51 spanning residues 29 to 170 is necessary and sufficient for the interaction with pUL7.

To confirm that pUL51 and pUL7 interact in the context of infection, HaCaT cells were infected with wild-type (WT) HSV-1 or HSV-1 lacking pUL51 expression (strain ΔpUL51), and cell lysates were subjected to immunoprecipitation using two different mouse monoclonal antibodies we generated for HSV-1 pUL51: 2B3 and 3D3. These antibodies recognize different epitopes within pUL51, with 2B3 binding to the C-terminal region (between residues 171 and 220) and 3D3 binding to the N-terminal region (between residues 1 and 170). Both pUL51 antibodies efficiently pulled down pUL51 from cells infected with WT HSV-1, and coprecipitation of pUL7 was observed with the 2B3 (C-terminal region-specific) pUL51 antibody, demonstrating that pUL51 and pUL7 interact in infected cells (Fig. 1C). Interestingly, the antibody that recognizes the N-terminal region of pUL51 (3D3) failed to coprecipitate pUL7, suggesting overlap in the binding sites on pUL51 for this antibody and for pUL7. Coimmunoprecipitation between pUL51 and pUL7 was detected when we used cells infected with HSV-1 and that expressed yellow fluorescent protein (YFP)-tagged pUL7, and we precipitated the complex with a GFP affinity resin (Fig. 1D).

### Direct interaction between pUL7 and pUL51.

**FIG 2:**
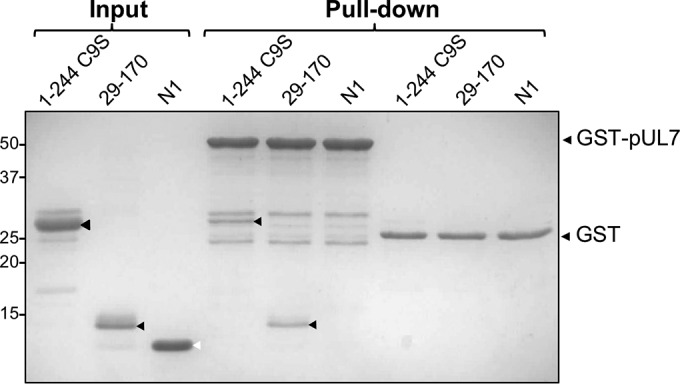
Direct interaction between pUL7 and pUL51. GST-tagged pUL7, His_6_-tagged full-length pUL51 with the cysteine at residue 9 mutated to serine to prevent aberrant disulfide bond formation (1–244 C9S), and truncated pUL51 (residues 29 to 170) proteins were expressed in E. coli and purified using affinity and size exclusion chromatography. Purified His_6_-tagged N1 protein from vaccinia virus was included as a negative control. GST-pUL7 or GST alone was captured on glutathione-magnetic beads and incubated with soluble pUL51 or N1, the beads were washed, and associated proteins were eluted for separation by SDS-PAGE. The black arrowheads denote full-length and truncated pUL51, while the white arrowhead denotes N1.

The observed interaction between pUL51 and pUL7 in transfected and infected cells could arise through direct binding of the two viral proteins or, alternatively, via an indirect interaction between both viral proteins and the same cellular protein or complex. To investigate whether there is a direct protein-protein interaction between pUL51 and pUL7, both proteins were expressed in Escherichia coli and purified. Both full-length pUL51 and the core pUL7-binding domain of pUL51 (residues 29 to 170) were fused to an N-terminal His_6_ tag, and pUL7 was fused to a C-terminal GST tag for affinity purification. Full-length pUL51 was mutated to change the cysteine at position 9 to a serine (C9S) to avoid the potential for protein aggregation by aberrant disulfide bond formation, cysteine 9 being the site of pUL51 palmitoylation in mammalian cells (13). GST pulldown assays were conducted using pUL7-GST or GST incubated with either full-length pUL51 (residues 1 to 244, C9S) or the core pUL7-binding domain (residues 29 to 170), or vaccinia virus protein N1 as a control. Clear coprecipitation of both pUL51 constructs was observed in the presence of pUL7-GST but not with GST alone (Fig. 2), demonstrating that pUL7 and pUL51 form a complex via a direct protein-protein interaction between pUL51 residues 29 to 170 and pUL7. Vaccinia virus N1 did not show any interaction with GST or pUL7-GST.

### Efficient expression of pUL7 and pUL51 is codependent in infected cells.

**FIG 3:**
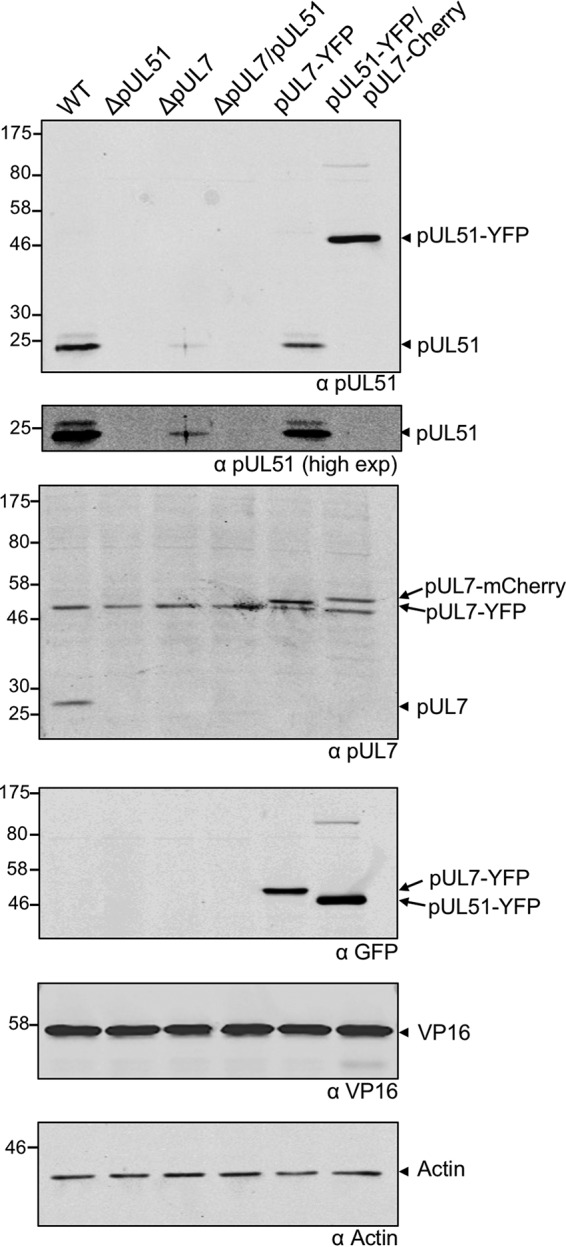
Protein expression profiles of mutant viruses. HaCaT cells were infected with WT or strain ΔpUL51, ΔpUL7, ΔpUL7/51, pUL7-YFP, and pUL51-YFP/pUL7-mCherry viruses and incubated for 16 h. Cells were lysed, separated by SDS-PAGE, and immunoblotted, probing for pUL51, pUL7, YFP, VP16, and actin.

To investigate the roles of pUL7 and pUL51 in infected cells, we created recombinant viruses lacking functional expression of either one or both of the proteins by engineering a stop codon cassette downstream of codon 15 in UL7 and codon 20 in UL51. We also generated recombinant viruses with enhanced YFP (EYFP) or mCherry fused to the C terminus of pUL7 or pUL51 to aid our investigations into the subcellular localization of these proteins in infected cells. Western blot analysis of protein expression levels in HaCaT cells infected by these viruses revealed that the presence of both components of the pUL7-pUL51 complex is necessary for stable expression of either protein, as levels of pUL7 and pUL51 were low or undetectable when the partner protein was missing (Fig. 3). These data also demonstrated that pUL7 or pUL51 tagged with EYFP or mCherry are expressed at similar levels as the wild-type proteins in infected cells.

### The pUL7-pUL51 complex localizes to focal adhesions.

**FIG 4:**
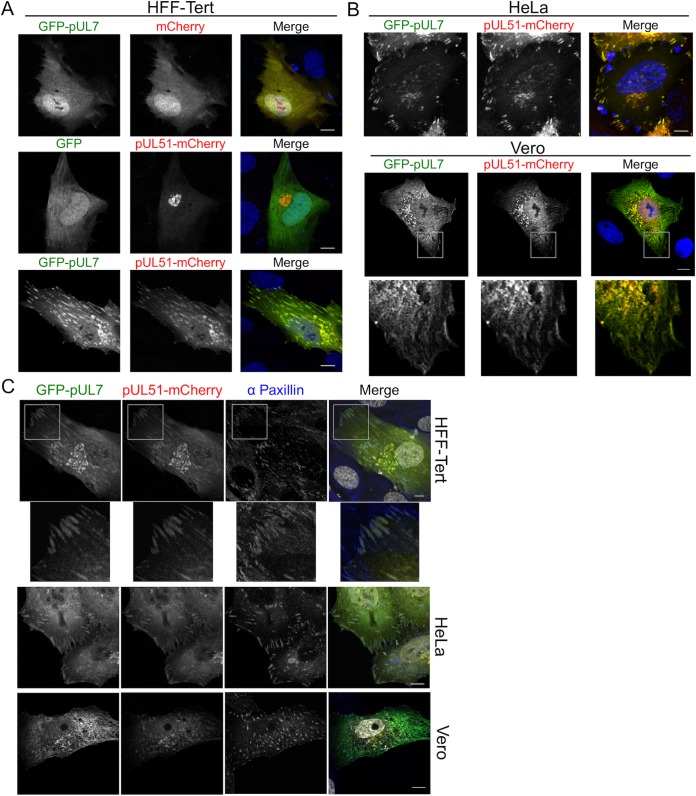
pUL7 and pUL51 localization in transfected cells. (A and B) HFF-Tert cells (A) and HeLa or Vero cells (B) were cotransfected with GFP-pUL7 plasmid or empty GFP plasmid control and pUL51-mCherry plasmid or empty mCherry plasmid control in various combinations. After 24 h, cells were fixed and imaged by confocal microscopy. (C) HFF-Tert, HeLa, or Vero cells were cotransfected with GFP-pUL7 and pUL51-mCherry plasmids and fixed after 24 h. Cells were then immunostained for paxillin and imaged by confocal microscopy. Nuclei were stained with DAPI (blue in panels A and B and grayscale in panel C). Bars, 10 μm.

To investigate the subcellular distribution of the pUL7-pUL51 complex in the absence of other HSV proteins, compared to the localization of pUL7 or pUL51 when expressed individually, we generated GFP-pUL7 (N-terminally tagged) and pUL51-mCherry (C-terminally tagged) expression plasmids and examined the localization of the fluorescent proteins in transfected HFF-Tert, HeLa, and Vero cells (Fig. 4). Unfused GFP and mCherry proteins were located throughout the cell in a diffuse pattern (Fig. 4A). GFP-pUL7 also demonstrated a diffuse expression pattern throughout the cell when coexpressed with unfused mCherry, suggesting that pUL7 does not contain specific localization signals nor interact with cellular membranes at a detectable level in these assays. When coexpressed with unfused GFP, pUL51-mCherry showed a juxtanuclear Golgi apparatus-like distribution, consistent with previous data showing that pUL51 localizes to the Golgi apparatus in a palmitoylation-dependent manner (13). When GFP-pUL7 and pUL51-mCherry were coexpressed, they demonstrated extensive colocalization, suggesting that pUL51 recruits pUL7 to intracellular membranes independently of other viral proteins. Interestingly, GFP-pUL7 and pUL51-mCherry localized not only to juxtanuclear compartments but also to striated structures at the cell periphery (Fig. 4A). Similar colocalization of GFP-pUL7 and pUL51-mCherry at juxtanuclear as well as peripheral striated structures was also observed in transfected HeLa and Vero cells, suggesting these localization patterns are not restricted to human fibroblasts (Fig. 4B). Immunostaining of cotransfected cells for the focal adhesion marker protein paxillin identified the GFP-pUL7/pUL51-mCherry-positive peripheral striated structures as focal adhesions in all three cell lines (Fig. 4C), although we noted that the focal adhesion structures were less obvious in Vero cells (see Discussion). No focal adhesion-like localization was observed for either GFP-pUL7 or pUL51-mCherry when expressed alone (Fig. 4A), suggesting that pUL7 and pUL51 efficiently associate with focal adhesions only when they are in complex with each other.

**FIG 5:**
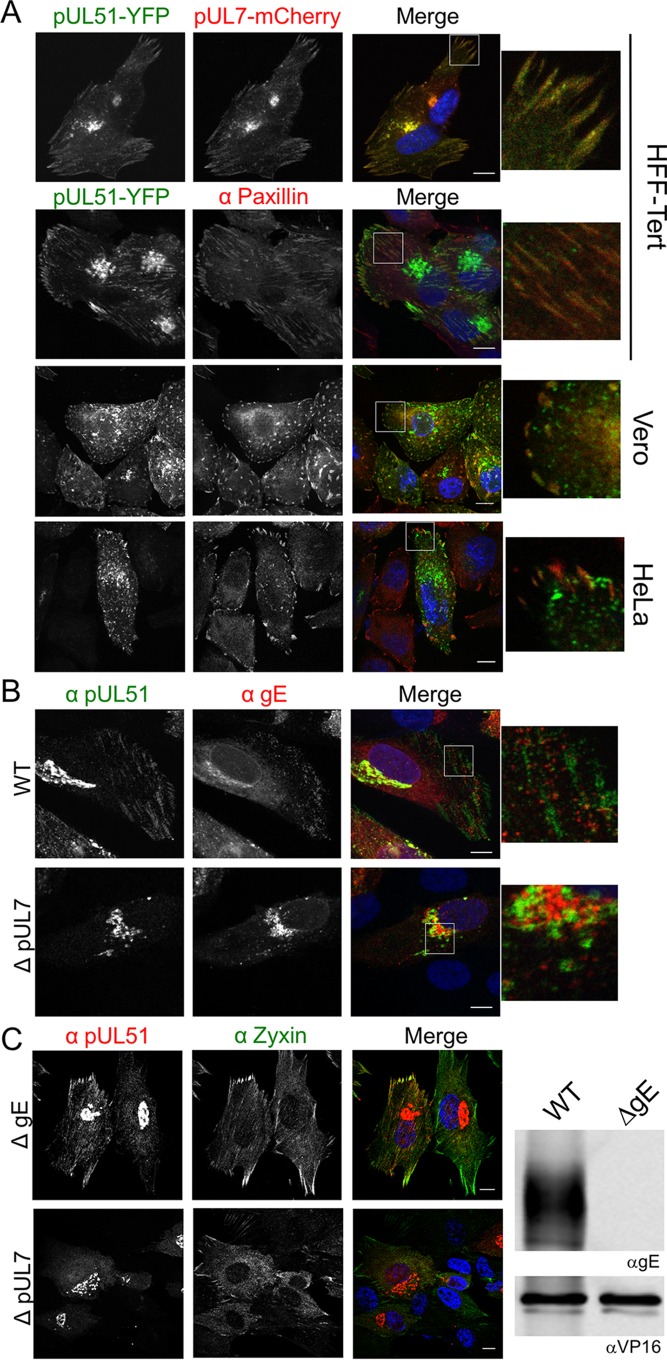
Localization of pUL51-pUL7 complexes in infected cells. (A) Cells were infected with pUL51-YFP/pUL7-mCherry virus and fixed after 8 h. The top panel shows colocalization of pUL51 with pUL7. Cells in lower panels were labeled for paxillin by using AlexaFluor 647 (far red channel). (B) HFF-Tert cells were infected with WT or strain ΔpUL7 viruses, fixed after 8 h, and labeled for pUL51 and gE. (C) HFF-Tert cells were infected with strain ΔgE or ΔpUL7 viruses, fixed after 8 h, and processed for immunostaining of pUL51 and zyxin. Lysates prepared from cells infected with WT or ΔgE viruses were analyzed by Western blotting using gE and VP16 specific antibodies (right panels). Nuclei were stained with DAPI (blue). Bars, 10 μm.

We next investigated pUL7 and pUL51 localization in infected cells. The monoclonal anti-pUL7 antibody we developed unfortunately did not provide reliable detection of pUL7 for immunofluorescence microscopy. Therefore, we used a recombinant virus where pUL51 is tagged at the C terminus with EYFP and pUL7 is tagged at the C terminus with mCherry. Analysis of cells infected with this double-fluorescent virus demonstrated that pUL7 and pUL51 localize to both focal adhesions and juxtanuclear compartments during infection in HFF-Tert, HeLa, and Vero cells (Fig. 5A). To confirm that fusion of pUL7 and pUL51 to mCherry and YFP did not influence their localization, HFF-Tert cells were infected with wild-type and mutant HSV-1 strains lacking fluorescent tags. While our antibody that recognizes pUL7 is not suitable for immunofluorescence microscopy, staining with the anti-pUL51 2B3 antibody clearly showed the presence of untagged pUL51 (and thus the pUL7-pUL51 complex) at peripheral striated focal adhesion structures (Fig. 5B and C). In cells infected with a recombinant virus lacking functional pUL7 expression (ΔpUL7 strain), no localization of pUL51 to the characteristic focal adhesion-like distribution could be detected, whereas pUL51 was still observed in a juxtanuclear compartment, albeit with a lower fluorescence intensity due to the reduced expression level of pUL51 in the absence of pUL7 (Fig. 5B). These data demonstrate that formation of the pUL7-pUL51 complex is necessary for focal adhesion localization of these proteins in infected cells. It has previously been demonstrated that pUL51 interacts with gE (15). In agreement with this, we observed areas of colocalization between pUL51 and gE in the juxtanuclear region and some peripheral punctate structures (potentially assembled virions or endocytic compartments where viral envelope and tegument proteins accumulate), and furthermore, this colocalization was independent of pUL7 expression (Fig. 5B). However, we observed no evidence of gE colocalizing with pUL51 at focal adhesion-like structures in HSV-1-infected cells, suggesting that the pUL7-pUL51 complex does not recruit gE to focal adhesions and that the observation of pUL7-pUL51 at these peripheral structures is not due to the presence of mature virions at focal adhesions (Fig. 5B). To further confirm focal adhesion localization using a different marker protein, zyxin, we infected cells with a virus lacking expression of gE (ΔgE strain) (Fig. 5C). A virus lacking gE was used to avoid potential cross-reactivity of the rabbit polyclonal anti-zyxin antibody with the HSV-1 gE-gI complex, an Fc receptor that binds rabbit immunoglobulins (20). pUL51 demonstrated good colocalization with zyxin in cells infected with the ΔgE strain but not in cells infected with the ΔpUL7 strain, further confirming association of the pUL51-pUL7 complex, but not pUL51 alone, with focal adhesions. We did not observe any obvious changes in pUL51 localization in ΔgE strain-infected cells compared to WT-infected cells, suggesting the interaction between gE and pUL51 is not necessary for localization of the pUL51-pUL7 complex to juxtanuclear structures or focal adhesions.

### pUL7-pUL51 functions as a complex during virus replication.

**FIG 6:**
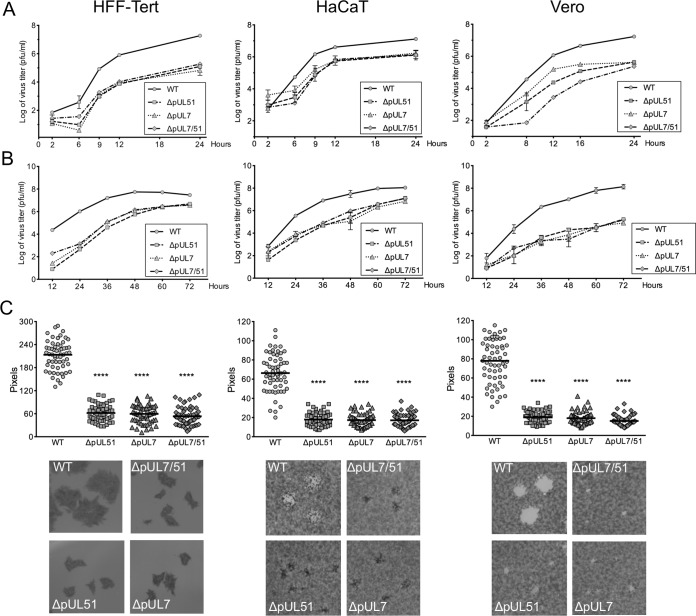
Growth curves and plaque sizes of deletion viruses in HFF-Tert, HaCaT, and Vero cells. (A and B) For single-step growth curves (A) and multistep growth curves (B), cells were infected at 5 PFU/cell or 0.01 PFU/cell, respectively. Samples were harvested at the indicated times, and titers were determined by plaque assay using Vero cells. Data are presented as means of duplicates of one representative experiment ± the standard error of the mean. (C) Monolayers of cells were infected with WT or deletion viruses for 72 h. After fixing, cells were either stained with toluidine blue (HaCaT and Vero) or incubated with gD-specific antibody followed by secondary HRP antibodies (HFF-Tert). Peroxidase activity was determined with DAB peroxidase substrate. Plates were scanned, and plaque diameters were measured in Adobe Photoshop. Data show plaque sizes on 60 plaques. *t* test results: ****, *P* < 0.0001. Representative plaques are shown in the bottom panels.

Previous studies have suggested a role for pUL51 in virus assembly (14–18). To investigate whether pUL51 needs to be in complex with pUL7 for its function during HSV-1 assembly, we performed single- and multistep growth analyses with recombinant virus strains lacking expression of functional pUL7 (ΔpUL7), pUL51 (ΔpUL51), or either protein (ΔpUL7/51). We observed that all three deletion virus strains demonstrated very similar growth kinetics to each other in three distinct cell lines (HFF-Tert, HaCaT, and Vero) (Fig. 6A and B). The lack of any additive or synergistic effect of deleting both pUL7 and pUL51 supports the hypothesis that these two proteins function as a complex. In single-step growth experiments, all three deletion viruses demonstrated similar ∼10- to 100-fold reductions in infectious titers at 24 h postinfection in each cell line tested, suggesting it is the pUL7-pUL51 complex that is required for efficient virus replication (Fig. 6A). Previous studies have demonstrated a role for pUL51 in cell-to-cell spread (15). In support of these observations, all three deletion virus strains were somewhat more attenuated in multistep replication than in single-step replication, in particular at earlier times (24 to 48 h) in HFF-Tert and HaCaT cells and throughout infection in Vero cells (Fig. 6B). Quantification of plaque sizes further supported a role of the pUL7-pUL51 complex in cell-to-cell spread, with significantly smaller plaques formed by all three deletion virus strains (Fig. 6C).

### The pUL7-pUL51 complex functions during HSV-1 assembly.

**FIG 7:**
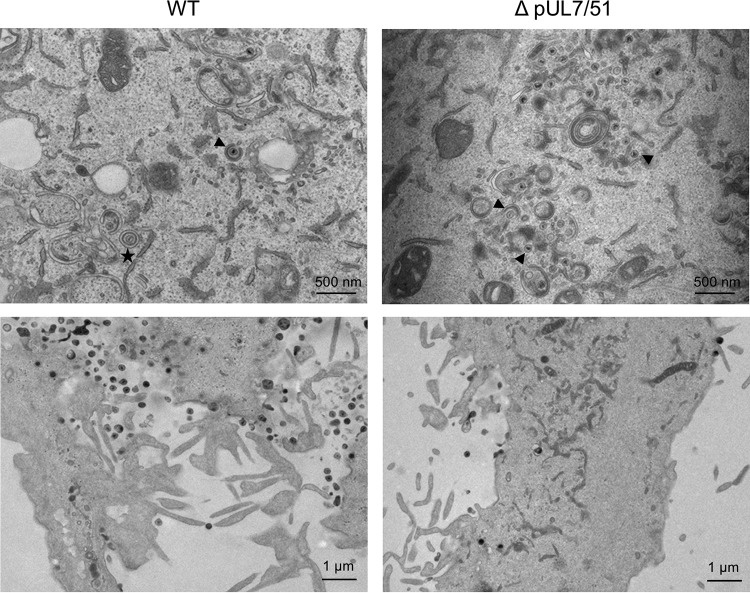
Electron micrographs of WT and strain ΔpUL7-51 virus-infected HFF-Tert cells. Cells were infected with viruses for 16 h and processed for transmission electron microscopy imaging. The top panels show assembly sites, and bottom panels show secreted viral particles. Arrowheads indicate representative nonenveloped or partially wrapped cytoplasmic nucleocapsids, and the star indicates an enveloped virion within a vesicle.

Our data show that the pUL7-pUL51 complex is required for efficient production of infectious viruses. To investigate which stage of the replication cycle is affected, we fixed HFF-Tert cells infected with WT or strain ΔpUL7/51 HSV-1 viruses at 16 h postinfection and processed samples for transmission electron microscopy imaging. In Fig. 7 we show representative cells; the images are focused on assembly sites (top panels) and on extracellular areas where secreted particles accumulate (bottom panels). The distribution of virus particles (unenveloped capsids and virions) in different cellular compartments (nuclear, cytoplasmic, and extracellular) were counted for at least 10 cells for each sample (Table 1). In WT HSV-1-infected cells, relatively few nucleocapsids were observed either without envelopes in the cytoplasm or in the process of being wrapped by membranes (secondary envelopment), whereas large numbers of virions were observed in the extracellular spaces, as expected for WT HSV-1 that undergoes rapid and efficient assembly and egress. However, in strain ΔpUL7/51-infected cells, many nonenveloped capsids were detected in the cytoplasm, of which the vast majority were in close proximity to membranes. Furthermore, fewer virions were observed in extracellular spaces for strain ΔpUL7/51-infected cells than for WT-infected cells. This suggests the major defect caused by the loss of a functional pUL7-pUL51 complex is an inhibition of secondary envelopment, similar to previously reported data for both PRV lacking pUL7 or pUL51 (11, 16) and HCMV lacking the pUL51 homologue pUL71 (18).

### pUL7-pUL51 stabilizes focal adhesions and maintains cell morphology in infected cells.

**FIG 8:**
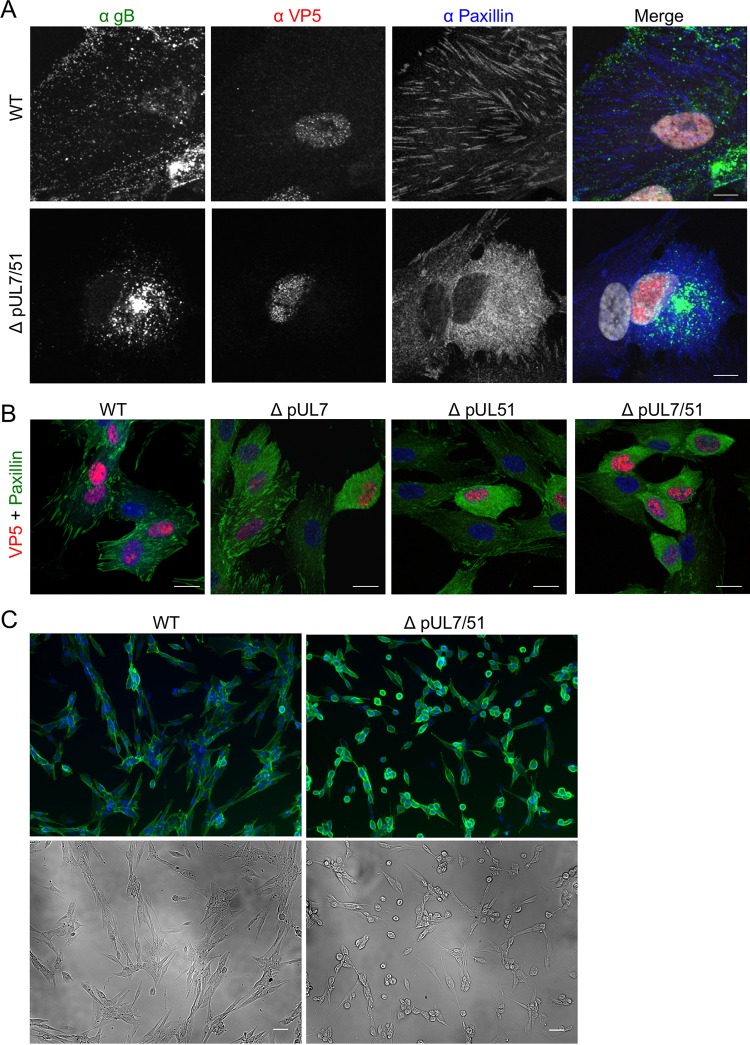
Protein localization and cell morphology in infected HFF-Tert cells. (A) Cells were infected with WT or ΔpUL7/51 strain virus and incubated for 8 h. After fixing, cells were labeled for gB, VP5, and paxillin. Bars, 10 μm. (B) Cells were infected with WT, ΔpUL7, ΔpUL51, or ΔpUL7/51 strain virus and incubated for 8 h. After fixing, cells were labeled for paxillin and VP5. Bars, 20 μm. (C) Cells were infected with either WT or ΔpUL7/51 strain viruses for 16 h, fixed, and then labeled for gD. Bars, 50 μm. Nuclei were stained with DAPI (grayscale in panel A and blue in panels B and C).

We next examined HFF-Tert cells infected with WT or strain ΔpUL7/51 HSV-1 by using confocal microscopy to investigate the effect of pUL7-pUL51 on focal adhesions. At 8 h postinfection with WT HSV-1, numerous focal adhesion structures could be readily observed at the basal plasma membrane, where the cell attaches to the coverslip. The envelope protein gB was visible in the juxtanuclear region as well as many small puncta distributed throughout the cytoplasm and at the cell periphery, where assembled virions accumulate. In the absence of pUL7-pUL51, the cytoplasmic and peripheral puncta were lost and virtually all gB accumulated in the juxtanuclear region (Fig. 8A). Similar effects were also observed for pUL51 when pUL7 was absent (Fig. 5B and C). Fewer peripheral gB-positive puncta likely indicates fewer enveloped virions in cytoplasmic carrier vesicles or at the cell surface because of reduced secondary envelopment in the absence of pUL7-pUL51. This does not appear to be due to a delay in virus replication, as similar levels of VP5 labeling were observed in the nucleus (Fig. 8A). Interestingly, we also observed a dramatic difference in cellular distribution of paxillin, with a predominantly diffuse cytoplasmic localization of this focal adhesion marker in cells infected with strain ΔpUL7/51 compared to the distinctive striated structures at the cell periphery in WT HSV-1-infected cells and uninfected cells. Furthermore, cells infected with strain ΔpUL7, ΔpUL51, or ΔpUL7/51 viruses were found to be generally smaller and more rounded than WT HSV-1-infected or uninfected cells, in addition to having more diffuse paxillin staining in the cytoplasm (Fig. 8B). The effect on cell morphology was more clearly observed at later times postinfection, when infected cells were fixed at 16 h postinfection, labeled with a gD-specific antibody, and cells were visualized at low magnification (Fig. 8C). These data demonstrated that strain ΔpUL7/51-infected cells more rapidly become rounded and detached than do WT HSV-1-infected cells. This suggests pUL7-pUL51 activity is required to maintain cell morphology, potentially by modulating focal adhesions that adhere the infected cells to the extracellular matrix and/or other cells.

## DISCUSSION

HSV-1 assembly is an intricate process, during which cytoplasmic nucleocapsids acquire a layer of tegument and a glycoprotein-containing envelope. The tegument is a complex structure composed of many different viral proteins with important roles in mediating virion assembly through interactions with the nucleocapsid and the envelope (5). An extensive network of interactions between tegument proteins has been identified that could control the virus assembly process (21, 22). However, this network of interactions between tegument proteins and their relative importance for virus assembly is still poorly understood. Key factors in controlling virion assembly are likely to be the major structural proteins, such as VP16, VP13/14, and VP22, as well as tegument proteins that directly interact with capsids, such as pUL36. For example, VP16 and VP22 interact with the cytoplasmic tails of viral envelope glycoproteins, and VP16 interacts with several other tegument proteins, including VP22 and pUL36, providing at least one mechanism to connect the envelope to the nucleocapsid via tegument proteins (23–26). Additionally, tegument proteins that directly bind to membranes via lipid anchors, such as pUL11 and pUL51, are likely to be important regulators of tegument association with membranes during secondary envelopment. Furthermore, tegument proteins that are conserved throughout the herpesvirus family are good candidates for mediating the core process of virus assembly. Here, we focused on the interaction between pUL7 and pUL51, two tegument proteins with homologues in all three herpesvirus subfamilies.

Our data demonstrate a robust and reproducible interaction between pUL7 and pUL51, as well as extensive colocalization of pUL7 and pUL51 when these proteins are expressed in transfected or infected cells (Fig. 1, 4, and 5), consistent with data reported recently (19). Importantly, using purified bacterially expressed proteins, we have demonstrated that pUL7 and pUL51 form a direct protein-protein interaction mediated by a core 142-amino-acid structural domain of pUL51 (amino acids 29 to 170) (Fig. 2). The formation of a complex between pUL7 and pUL51 appears to be important for the stability of both of the proteins in infected cells, as expression of either protein is poor when the partner protein is absent (Fig. 3). Additionally, a virus lacking expression of both pUL7 and pUL51 shows very similar replication kinetics to viruses lacking either pUL7 or pUL51 individually (Fig. 6). Taken together, our results suggest that pUL51 and pUL7 function as a complex and, when expressed alone, both of these proteins are less stable, perhaps due to being more prone to misfolding or aggregation and thus being directed for degradation.

While not essential for HSV-1 replication, the pUL7-pUL51 complex is clearly important for efficient assembly of HSV-1; virus lacking this complex produces lower infectious titers at the end of a single cycle of replication (Fig. 6), and cells infected with strain ΔpUL7/51 HSV-1 accumulate partially enveloped nucleocapsids in the cytoplasm (Fig. 7 and Table 1). This suggests that the pUL7-pUL51 complex may be important for recruitment of the other tegument proteins to secondary envelopment sites and/or the coordinated assembly of tegument-envelope and tegument-capsid interactions necessary for efficient HSV-1 assembly. Interestingly, a recent report demonstrated that pUL14, another conserved tegument protein, interacts with pUL51 in HSV-1-infected cells, and inhibition of this interaction also caused defects in secondary envelopment (27). Furthermore, these studies demonstrated that deletion of pUL14 had similar effects on HSV-1 replication and assembly as the deletion of pUL51, suggesting that these two proteins may also function as a complex during virus assembly. It will be interesting to investigate whether the pUL14-pUL51 interaction is also direct, the impact of pUL7 on this interaction, and whether these three conserved tegument proteins form a higher-order complex.

**TABLE 1:** Quantification of electron microscopy data

Virus strain	No. of particles (% of total) in location					
	Capsids in nucleus	Enveloped virions in perinuclear space	Unenveloped capsids in cytoplasm	Membrane-associated unenveloped capsids in cytoplasm	Enveloped virions in cytoplasm	Extracellular virions
WT	1138 (68.6)	9 (0.5)	3 (0.2)	24 (1.4)	31 (1.9)	455 (27.4)
ΔUL7/ΔUL51	469 (48.8)	11 (1.1)	23 (2.4)	412 (42.9)	20 (2.1)	26 (2.7)

Immunofluorescence experiments performed using cells infected with HSV-1 or transfected with fluorescently tagged pUL51 and pUL7 constructs revealed two distinct locations for the pUL51-pUL7 complex within cells: juxtanuclear compartments and focal adhesions at the periphery of the cell (Fig. 4 and 5). While localization of pUL51 (and pUL7 when coexpressed with pUL51) at juxtanuclear compartments is consistent with findings from previous studies (13, 19), a recent study investigating pUL51 and pUL7 localization in Vero cells did not report any focal adhesion-like localization (19). However, it is important to note that focal adhesions are difficult to observe in Vero cells. We found that careful imaging with confocal microscopy of the basal membrane of cells that were attached to the coverslip was required in order to detect focal adhesions and the association of pUL7-pUL51 with these peripheral structures in Vero cells.

Localization of pUL51 to juxtanuclear compartments is independent of pUL7 expression, presumably being driven by a palmitoylation-dependent membrane association of pUL51 (13), while localization of pUL7 to these compartments relies on the presence of pUL51, as previously reported (19). HSV-1 structural proteins are known to accumulate at juxtanuclear compartments, and virion assembly occurs at membranes that contain cellular *trans*-Golgi network and endosome marker proteins (5, 28). It therefore seems likely that pUL51 and pUL7 localized at these juxtanuclear compartments act in concert to aid virus assembly and secondary envelopment, perhaps in conjunction with pUL14 (27).

Localization of pUL7 and pUL51 to focal adhesions requires formation of the pUL7-pUL51 complex, as neither protein is able to localize to focal adhesions in the absence of the other. Focal adhesions are contact sites between the extracellular matrix and the cytoplasm. They are complex and dynamic structures that contain over a hundred cellular proteins and play important signaling roles in response to a variety of extracellular stimuli (29, 30). Focal adhesions can regulate many fundamental cellular processes, including cell division and apoptosis (31). Importantly, they also play a crucial role in cell attachment and movement (32). In this study, we observed that cells infected with strain ΔpUL7/51 HSV-1 had less-pronounced focal adhesions and rounded up more quickly than cells infected with wild-type HSV-1 (Fig. 8), suggesting that the pUL51-pUL7 complex stabilizes focal adhesions to maintain cell shape during HSV-1 infection. Interestingly, a similar phenotype was observed when epithelial cells were infected with VZV lacking the UL51 homologue, ORF7 (33), suggesting that the maintenance of cell attachment is a conserved function of pUL51 in alphaherpesviruses. HSV-1 pUL51 has previously been suggested to be involved in cell-to-cell spread of virus due to the small-plaque phenotype of pUL51 deletion viruses (15). Our data suggest it is the activity of the pUL7-pUL51 complex that is important for any function of these tegument proteins in cell-to-cell spread, as we observed that removal of either pUL7, pUL51, or both proteins from HSV-1 led to equivalently dramatic reductions in plaque sizes (Fig. 6). We propose that the pUL7-pUL51 complex may be able to enhance virus cell-to-cell spread by stabilizing focal adhesions during HSV-1 infection. To date there have been few investigations into the role of focal adhesions in HSV infection, with most studies focusing on the role of focal adhesions and integrin signaling during virus entry (34, 35). During infection, viruses reprogram cell behavior to favor viral replication and spread. In the case of HSV-1, infected cells can form long filopodium-like projections that are thought to aid transfer of newly assembled viruses to neighboring cells (36). Focal adhesions are necessary for lamellipodium and filopodium formation (37), so destabilization of focal adhesions during infection could impede viral spread due to loss of filopodium-like projections. In addition, focal adhesion-mediated attachment to the extracellular matrix may be important for maintaining cell-cell contacts, thereby maintaining the proximity of infected cells to uninfected neighboring cells, which could enhance the rate of virus spread. At present, we do not know the mechanism by which pUL7-pUL51 localizes to focal adhesion complexes. We are currently trying to address this question by identifying cellular binding partners for the pUL7-pUL51 complex.

A previous study reported that pUL51 binds the HSV-1 glycoprotein gE (15). Work with HSV-1 lacking gE expression also demonstrated a small-plaque phenotype similar to pUL7 or pUL51 deletion viruses (38, 39), which could suggest that gE and pUL7-pUL51 have a related function during cell-to-cell spread. Given that gE appears to travel to virion assembly compartments via the plasma membrane (40), it is conceivable that interaction with gE facilitates recruitment of pUL7-pUL51 to focal adhesion complexes at the plasma membrane. However, we could not detect gE at focal adhesions in infected cells, and deletion of gE did not appear to affect recruitment of pUL7-pUL51 to focal adhesions, suggesting that focal adhesion-associated functions of pUL7-pUL51 are independent of any interaction with gE.

In conclusion, our study demonstrates that pUL7 and pUL51 form a direct protein-protein interaction and this interaction is important for the integrity of both proteins during virus infection. Furthermore, our data suggest that pUL7 and pUL51 function as a complex to promote efficient secondary envelopment and, potentially, virus spread. Importantly, we have identified a novel function of the pUL7-pUL51 complex in the regulation of focal adhesion stability and maintenance of cell morphology during infection, which opens up several new areas for investigation into these conserved herpesvirus tegument proteins. The role of pUL7 and pUL51 in virus assembly appears to be conserved among Herpesviridae, as defects in secondary envelopment have been reported for PRV and HCMV lacking the relevant homologues (11, 16–18). It will be interesting to investigate whether the interaction between pUL51 and pUL7 is conserved in other herpesvirus family members and whether such complexes localize to focal adhesions, as an ability to stabilize focal adhesions and thus maintain cell morphology may significantly influence the spread of viruses that are transmitted primarily by direct cell-to-cell transfer.

## MATERIALS AND METHODS

### Cell lines and viruses.

293T cells (ATCC), Vero cells (ATCC), HaCaT (41), telomerase immortalized human foreskin fibroblasts (HFF-Tert) (42), and HeLa cells (ATCC) were grown in DMEM supplemented with 10% fetal bovine serum (FBS), 2 mM glutamine, 100 U ml^−1^ penicillin, and 100 μg ml^−1^ streptomycin. The KOS strain of HSV-1, reconstituted from a cloned bacterial artificial chromosome, was used as WT virus (43). All pUL7 and pUL51 deletion and tagged viruses and the gE deletion virus were constructed using the bacterial artificial chromosome (BAC)-cloned KOS strain HSV-1 (43), using the primers shown in Table 2 and the two-step Red recombination technique (44). To delete functional pUL7 and pUL51 expression (strains ΔpUL7 and ΔpUL51), three tandem in-frame stop codons were inserted in place of codons 16 to 18 of UL7 and codons 21 to 23 of UL51. A double-deletion virus (strain ΔpUL7/51) was generated by inserting the stop codons into UL51 using the strain ΔpUL7 virus BAC-cloned genome. To fluorescently tag pUL7, EYFP (A206K) or mCherry coding sequences were inserted in frame at the 3′ end of the UL7 gene immediately upstream of the stop codon. To fluorescently tag pUL51, the EYFP (A206K) coding sequence was inserted at the 3′ end of the UL51 gene immediately upstream of the stop codon. To delete functional gE expression, three tandem in-frame stop codons were inserted in place of codons 21 to 23 of US8.

**TABLE 2:** Oligonucleotide primers

Name	Sequence (5′–3′)	Details
COL450	CCGCACCTCGCCCTGGCCCATCTCGAGCAGCTCCGTGTGTTTTGGGTCAAGTGAGCAAGGGCGAGGAG	Forward primer for inserting in-frame EYFP(A206K) at C terminus of pUL51
COL451	CTCCATCCCACAAACACAAAACACACGGGTTGGATGAAAACACGCATTTACTTGTACAGCTCGTCCATG	Reverse primer for inserting in-frame EYFP(A206K) at C terminus of pUL51
COL456	TTTCGGAGACCCCAAAACGACAGACGTCGTCGCTGTTTTATCAGTTTTGTGTGAGCAAGGGCGAGGAG	Forward primer for inserting in-frame EYFP(A206K) or mCherry at C terminus of pUL7
COL457	ATCCGTCGGGAGGCCACAGAAACAAAACCGGGTTTATTTCCTAAAATTCACTTGTACAGCTCGTCCATG	Reverse primer for inserting in-frame EYFP(A206K) or mCherry at C terminus of pUL7
COL496	CGCCGCGACGGCCGACGATGAGGGGTCGGCCGCCACCATCTAGTAATGAATTCTCGCCGGGGACCGCAGCCTGAGGATGACGACGATAAGTAGGG	Forward primer for replacing codons 16–18 of UL7 with three stop codons
COL497	ATCGCCTCGGCCGCCTCGACCAGGCTGCGGTCCCCGGCGAGAATTCATTACTAGATGGTGGCGGCCGACCCCTCAACCAATTAACCAATTCTGATTAG	Reverse primer for replacing codons 16–18 of UL7 with three stop codons
COL498	TATATGTGGCTGGGGAGCGCGCCCCGAGGAACAATATGAGTAGTGATAAGGATCCGTTCCGCCCTCGGAGGCGGAAGGATGACGACGATAAGTAGGG	Forward primer for replacing codons 21–23 of UL51 with three stop codons
COL499	GGGCCTCCTGCAGCCGCGGCTCCGCCTCCGAGGGCGGAACGGATCCTTATCACTACTCATATTGTTCCTCGGGGCCAACCAATTAACCAATTCTGATTAG	Reverse primer for replacing codons 21–23 of UL51 with three stop codons
COL523	GGGGTTTCTTCTCGGTGTTTGTGTTGTATCGTGCTTGGCGTAGTGATAAGCTTCGTCCTGGAGACGGGTGAGTAGGATGACGACGATAAGTAGGG	Forward primer for replacing codons 21–23 of US8 with three stop codons
COL524	AACGAAACGTCCTCGCCGACACTCACCCGTCTCCAGGACGAAGCTTATCACTACGCCAAGCACGATACAACACCAACCAATTAACCAATTCTGATTAG	Reverse primer for replacing codons 21–23 of US8 with three stop codons

### Reagents.

Monoclonal antibodies to pUL51 and pUL7 were generated by inoculating female BALB/c mice with HSV-1 using ear scarification, followed by immunization with purified pUL51 or pUL7 protein and a virus protein boost 1 month later. After 3 days, spleens were harvested and hybridomas were generated as described previously (45). Hybridomas secreting antibodies specific to pUL51 or pUL7 were identified by immunofluorescence screening and enzyme-linked immunosorbent assays and cloned by limiting dilution. Two antibodies specific to pUL51 were used in this work: antibody 3D3 (used for Western blotting and immunoprecipitation) recognizes an epitope contained within amino acids 1 to 170 of pUL51; antibody 2B3 (used for immunofluorescence and immunoprecipitation) recognizes an epitope contained within amino acids 171 to 220 of pUL51. Monoclonal antibodies to other viral proteins were described previously: gD (LP2 [46]), gB (CB24 [47]), gE (3114 [48]), VP16 (LP1; Abcam, ab110226 [49]), pUL36 (CB4 [50]), and VP5 (LP12 [51]). Anti-paxillin (610051) was from BD Biosciences, anti-zyxin (ab71842) and anti-actin (AC-40) were from Abcam, anti-GFP (JL8, which also recognizes YFP) was from Clontech, and anti-GST (sc459) was from Santa Cruz Biotechnology. All Western blot assay secondary antibodies were from Li-Cor, and the fluorescent secondary Alexa Fluor antibodies were from Molecular Probes. Anti-mouse horseradish peroxidase (HRP)-conjugated antibodies were from Dako. The ImmPACT diaminobenzidine (DAB)-peroxidase (HRP) substrate kit was from Vector (SK4105). Glutathione-Sepharose 4B beads were from GE Life Sciences, and protein A/G Plus-agarose beads were from Santa Cruz Biotechnology (sc2003). Transfections of 293T cells were performed with polyethylenimine. Other cell lines were transfected with TransIT-LT1 transfection reagent (Mirus).

All procedures for mouse immunizations were approved by the University of Cambridge ethical review board and by the UK Home Office under the 1986 Animal (Scientific Procedures) Act (project license 80/2538).

### GST pulldown assay from mammalian cell lysates.

Full-length and fragments of UL51 and full-length UL7 were cloned into GFP (pEGFPC2) and GST (pCAG-GST-ENX) expression plasmids, yielding clones of pUL51 or pUL7 with GFP or GST fused to the N terminus. 293T cells were seeded into six-well dishes and transfected with the GST- and GFP-harboring plasmids by using polyethylenimine. At 48 h posttransfection, cells were washed with phosphate-buffered saline (PBS) and lysed with 500 μl of lysis buffer (50 mM Tris [pH 7.4], 150 mM NaCl, 5 mM EDTA, 5% [vol/vol] glycerol, 1% Triton X-100) supplemented with a protease inhibitor cocktail (Roche). Lysates were clarified at 17,000 × *g* for 10 min at 4°C, and then 450 μl of lysate was incubated with glutathione-Sepharose 4B at 4°C on a rotating wheel for 4 h. Sepharose beads were harvested at 2,400 × *g* for 3 min at 4°C and washed five times with lysis buffer. Bound protein complexes were eluted by heating in SDS-PAGE sample buffer at 98°C for 5 min and analyzed by Western blotting using antibodies specific for GST and GFP. Input lanes represent ∼1.6% of total lysate, and pulldown lanes represent material precipitated from ∼18% of total lysate.

### Immunoprecipitation.

HaCaT cells were infected (or mock infected) with either WT or strain ΔpUL51 at 3 PFU/cell. After 16 h, cells were washed with ice-cold PBS and lysed in 10 mM Tris (pH 7.5), 150 mM NaCl, 0.5% NP-40, 1% EDTA-free protease inhibitor cocktail (Sigma-Aldrich), and centrifuged to remove the insoluble fraction. For pUL51 immunoprecipitation, lysates were precleared by incubation with equilibrated protein A/G beads for 30 min at 4°C, supernatant was removed and incubated with anti-pUL51 antibody (3D3 or 2B3) for 2 h at 4°C, and equilibrated protein A/G beads were added and incubated for a further 1 h. For YFP immunoprecipitation, lysates were incubated with equilibrated GFP-Trap agarose beads (ChromoTek) for 1 h at 4°C. Beads were washed three times with ice-cold wash buffer (10 mM Tris [pH 7.5], 150 mM NaCl, 0.5 mM EDTA) and then boiled in 2× SDS-PAGE loading buffer to elute protein for SDS-PAGE and immunoblotting. For anti-pUL51 immunoprecipitation samples, input lanes represent ∼1.6% of total lysate, and each pulldown lane represents material precipitated from ∼13.7% of total lysate. For GFP-Trap samples, input lanes represent ∼1.6% of total lysate and pulldown lanes represent material precipitated from ∼22.5% of total lysate.

### Recombinant protein expression.

Full-length UL51 from HSV-1 KOS was cloned into pOPTH for bacterial expression with an N-terminal MetAlaHis_6_ tag, and residue Cys9 was mutated to serine by QuikChange mutagenesis (Agilent Technologies). A truncated UL51 construct expressing only the core pUL7-binding domain (amino acids 29 to 170) in the same plasmid was generated by QuikChange mutagenesis. The gene sequence encoding full-length pUL7 from HSV-1 KOS was optimized for codon usage in Escherichia coli, synthesized (GeneArt), and cloned into pOPTn3G, a modified pOPT plasmid (52) that appends a GST tag preceded by a human rhinovirus 3C protease cleavage site to the C terminus of the expressed protein.

His_6_-pUL51(C9S) and His_6_-pUL51(29–170) were expressed in E. coli BL21(DE3) pLysS (Novagene). pUL7-GST was expressed in E. coli Rosetta2(DE3) pLysS (Novagene). Bacterial cultures were grown at 37°C in 2× tryptone-yeast extract medium with appropriate antibiotic selection to an optical density at 600 nm of 0.8 to 1.0, whereupon cultures were cooled to 22°C and protein expression was induced by the addition of 0.2 mM isopropyl β-d-thiogalactopyranoside. Cells were harvested 16 h postinduction by centrifugation, and bacterial pellets were stored at −80°C.

### Recombinant protein purification and pulldown assays.

To purify His_6_-pUL51(C9S) or His_6_-pUL51(29–170), cells were thawed in lysis buffer (20 mM Tris [pH 7.5], 500 mM NaCl, 20 mM imidazole [pH 7.5], 1.4 mM β-mercaptoethanol, and 0.05% Tween 20 and supplemented with 400 U bovine DNase I [Sigma-Aldrich] and 200 μl EDTA-free protease inhibitor cocktail [Sigma-Aldrich] per 2 liters of bacterial culture), and lysed at 24,000 lb/in^2^ using a TS series cell disrupter (Constant Systems). Lysates were centrifuged at 40,000 × *g* for 30 min at 4°C. The cleared lysate was incubated for 1 h at 4°C with Ni^2+^-nitrilotriacetic acid–agarose resin (Qiagen) that had been preequilibrated with wash buffer (20 mM Tris [pH 7.5], 500 mM NaCl, 20 mM imidazole [pH 7.5]), the resin was washed, and bound protein was eluted in elution buffer (20 mM Tris [pH 7.5], 500 mM NaCl, 250 mM imidazole [pH 7.5]). Size exclusion chromatography was performed using a Superdex 75 16/600 column (GE Healthcare) equilibrated in gel filtration buffer (20 mM Tris [pH 7.5], 200 mM NaCl, 1 mM dithiothreitol [DTT]). Purified protein was concentrated using centrifugal concentrators (Millipore), and 100-μl aliquots were snap-frozen in liquid nitrogen for storage at −80°C. GST and pUL7-GST were purified following the same general procedure, using glutathione-Sepharose 4B immobilization resin (GE Life Science) and the following buffers: lysis buffer, 20 mM Tris (pH 7.5), 300 mM NaCl, 1.4 mM β-mercaptoethanol, 0.05% Tween 20, supplemented with 400 U bovine DNase I and 200 μl EDTA-free protease inhibitor cocktail per 2 liters of bacterial culture; wash buffer, 20 mM Tris (pH 7.5), 300 mM NaCl, 1 mM DTT; elution buffer, wash buffer supplemented with 25 mM reduced glutathione (Sigma-Aldrich); gel filtration buffer, 20 mM Tris (pH 7.5), 200 mM NaCl, 1 mM DTT. Vaccinia virus protein N1-His_6_ with a C40S mutation to stop protein aggregation was purified as described previously (53).

For pulldown assays using purified proteins, magnetic glutathione beads (Pierce) were equilibrated in pulldown buffer (20 mM Tris [pH 7.5], 200 mM NaCl, 0.1% Nonidet P-40, 1 mM DTT, 1 mM EDTA) and then incubated with 2.5 μM bait protein (UL7-GST or GST) for 15 min at 4°C. Beads were washed with pulldown buffer and incubated with 20 μM prey protein [His_6_-pUL51(C9S), His_6_-pUL51(29–170), or N1-His_6_] for 1 h at 4°C. Unbound protein was removed, the beads were washed four times with pulldown buffer, and bound protein was eluted in pulldown buffer supplemented with 50 mM reduced glutathione. Samples were resolved by SDS-PAGE and visualized using Coomassie stain.

### Immunofluorescence microscopy.

Cells for imaging were grown in 24-well plates on coverslips. Samples were fixed with 4% (vol/vol) electron microscopy-grade formaldehyde (Polysciences) for 10 min and then permeabilized with 0.1% Triton for 5 min. Cells were then incubated with blocking buffer (2% [vol/vol] FBS in PBS), with the addition of 100 μg/ml human IgG when rabbit primary antibodies were used, for 30 to 60 min and then with primary antibodies. Secondary antibodies used were anti-rat, anti-rabbit, and anti-mouse isotype-specific AlexaFluor 488, 568, or 647. Coverslips were mounted on microscope slides by using ProLong Gold antifade reagent with 4′,6-diamidino-2-phenylindole (DAPI; Thermo Fisher). All images were acquired with a confocal microscope (Zeiss LSM780 or Leica SP5).

For cell morphology assays, HFF-Tert cells grown in 6-well dishes were infected at 5 PFU/cell and incubated for 16 h. Cells were fixed with 4% (vol/vol) formaldehyde for 20 min, washed with PBS, and then incubated with anti-gD (LP2). Samples were then washed in PBS, incubated with AlexaFluor 488 secondary antibody, incubated with PBS containing DAPI, washed with PBS, and then stored in PBS. Images were acquired using the 10× lens of an Olympus IX81 wide-field fluorescence microscope and the Image-Pro Plus software (Media Cybernetics).

### Viral growth curves and plaque assays.

HFF-Tert, HaCaT, or Vero cells were grown in 24-well plates and infected with WT or deletion viruses at 5 PFU/cell for single-step growth curves or 0.01 PFU/cell for multistep growth curves. After 1 h of infection, unabsorbed viruses were inactivated with an acid wash (40 mM citric acid, 135 mM NaCl, 10 mM KCl; pH 3.0) for 1 min at room temperature. Cells were then washed with PBS three times and grown in culture medium. At various time points postinfection, samples were harvested by freezing at −70°C. Freeze-thawing was repeated twice, and virus titers were determined by plaque assays on Vero cells. For plaque size quantification, HaCaT, Vero, and HFF-Tert cells were grown as monolayers on 6-well plates. Tenfold serial dilutions of viruses were prepared and added to cells for 1 h. Wells were then overlaid with medium containing 0.6% carboxymethyl cellulose and 2% (vol/vol) FBS. After 72 h, cells were fixed with formal saline and then stained with 0.1% toluidine blue solution. For HFF-Tert cells, which do not form clear plaques, cell monolayers were labeled with anti-gD antibody (LP2) followed by secondary antibody conjugated to horseradish peroxidase. Plaques were revealed using DAB peroxidase substrate following the manufacturer's instructions (Vector SK4105). All plaques were scanned, and diameters were determined in pixels using Adobe Photoshop.

### Electron microscopy.

HFF-Tert cells were infected at 5 PFU/cell and fixed at 16 h postinfection in 0.5% glutaraldehyde in 200 mM sodium cacodylate buffer for 30 min, washed in buffer, and secondarily fixed in reduced 1% osmium tetroxide, 1.5% potassium ferricyanide for 60 min. The samples were washed in distilled water and stained overnight at 4°C in 0.5% magnesium uranyl acetate, washed in distilled water, and dehydrated in graded ethanol. The samples were then embedded flat in the dish in epon resin. Resin-filled stubs were placed on embedded cell monolayers and polymerized. Ultrathin sections (typically 50 to 70 nm) were cut parallel to the dish and examined in a FEI Tecnai electron microscope with a charge-coupled-device camera for image acquisition. For quantification, the location and type of all virus particles in 10 to 11 cells for each sample were enumerated. Data represent the sum of particles in each category from all cells and the percentage of the total for each condition.
